# A Nation-Wide Cancer Registry-Based Study of Adenosquamous Carcinoma in Taiwan

**DOI:** 10.1371/journal.pone.0139748

**Published:** 2015-10-07

**Authors:** Yuan-Tzu Lan, Kuo-Hung Huang, Chien-An Liu, Ling-Chen Tai, Ming-Huang Chen, Yee Chao, Anna Fen-Yau Li, Shih-Hwa Chiou, Yi-Ming Shyr, Chew-Wun Wu, Wen-Liang Fang

**Affiliations:** 1 Division of Colon & Rectal Surgery, Department of Surgery, Taipei Veterans General Hospital, Taipei, Taiwan; 2 Division of General Surgery, Department of Surgery, Taipei Veterans General Hospital, Taipei, Taiwan; 3 Department of Radiology, Taipei Veterans General Hospital, Taipei, Taiwan; 4 Division of Hematology and Oncology, Department of Medicine, Taipei Veterans General Hospital, Taipei, Taiwan; 5 Department of Pathology, Taipei Veterans General Hospital, Taipei City, Taiwan; 6 School of Medicine, National Yang-Ming University, Taipei, Taiwan; 7 Institute of Clinical Medicine, School of Medicine, National Yang-Ming University; 8 Department of Medical Research and Education, Taipei Veterans General Hospital, Taipei City, Taiwan; 9 Institute of Pharmacology, National Yang-Ming University, Taipei City, Taiwan; Kaohsiung Chang Gung Memorial Hospital, TAIWAN

## Abstract

**Background:**

Adenosqamous carcinoma (ASC) is a rare disease involving various organs, yet there are no large-scale population-based comparative studies on ASC among different organs.

**Methods:**

The incidence and overall survival of ASC among various organs in cases diagnosed in Taiwan from January 1, 2003 to December 31, 2010 were calculated and compared using data from the Taiwan Cancer Registry (TCR). The various organs were classified and divided into three different systems: the female reproductive, respiratory, and alimentary systems. Survival analysis were also compared among 30,850 patients diagnosed as ASC, adenocarcinoma (AC) or squamous cell carcinoma (SCC) in organs with frequent ASC.

**Results:**

During the study period, a total of 576 ASC cases were diagnosed in Taiwan. The most common primary system was respiratory (73.8%), followed by alimentary (16.2%) and female reproductive (10%). The overall survival were significantly higher for cases involving the female reproductive system, followed by the respiratory and alimentary systems (*P* = 0.016). The median overall survival were worse in males than females for cases involving the respiratory system (22.4 vs. 31.8 months, *P* = 0.044). Multivariate analysis showed that age≧65, more advanced T and N categories were independent unfavorable prognostic factors of overall survival in ASC. ASC histology is an independent unfavorable prognostic factor compared with AC and SCC.

**Conclusions:**

ASC at an old age and more advanced T and N categories were found to be associated with a poor prognosis.

## Introduction

The histologic features of adenosquamous carcinoma (ASC) include an infiltrating neoplasm with solid and glandular components; squamous differentiation is evidenced by individual cell keratinization, intercellular bridges, keratin pearl formation and/or dyskeratosis, and glandular differentiation by various-sized gland formations and intracellular and intraluminal mucin]. To qualify as ASC, both adenocarcinoma and squamous cell carcinoma components must be present. ASC has aggressive clinicopathological features and a poorer prognosis than typical adenocarcinomas.

ASC is a rare variant of metaplastic carcinoma in various organs, and the prognostic role of ASC histology is different among organs. For examples, ASCs of breast cancer [2] are often low grade and characterized by a favorable prognosis. In cervical cancer, it remains controversial whether the ASC histological subtype is an independent prognostic factor. Although some studies do not make a distinction between adenocarcinoma and ASC and include ASC as a subtype of adenocarcinoma when evaluating the outcomes of cervical cancer [3–6], others studies report that patients with ASC have a poorer prognosis than those with adenocarcinoma [7–9].

In lung cancer, ASC is an unusual and aggressive form of non-small cell lung carcinoma and accounts for 0.4–4% of all lung cancers [10–16]. In head and neck cancer, ASC is a rare malignancy and the tumor behavior is extremely aggressive, with 80% of patients developing metastases [17,18].

In the alimentary system, esophageal ASC is a rare disease, representing 0.92% of all esophageal carcinomas; the prognosis is poorer than that of esophageal squamous cell carcinoma but similar to that for poorly differentiated SCC patients [19,20]. ASC in gastric cancer is very rare, comprising <0.5% of all gastric malignancies [21–24]; most of the reported cases are in Asians, and the disease is associated with a poor prognosis. ASC in colorectal cancer is as rare as 0.09% and is associated with higher overall and colorectal-specific mortality compared with adenocarcinoma [25]. With regard to the liver, most malignant primary tumors are hepatocellular carcinoma and cholangiocarcinoma. ASC of the liver which is considered to be a variant of cholangiocarcinoma is very rare and has a poor prognosis [1]. In the extrahepatic bile duct, ASC accounts for 2–5% of cases, with a worse survival than in cases of adenocarcinoma [26]. These results suggested a very low incidence rate of ASC in various organs.

This study analyzed the incidence rate and the overall survival rate of ASCs in Taiwan using data from the Taiwan Cancer Registry (TCR) from 2003 to 2010. Furthermore, we also performed the survival analysis for patients diagnosed as AC, SCC or ASC in organs frequent with ASC. To our knowledge, this is the first nation-wide cancer registry-based study of ASCs.

## Materials and Methods

The ASC cases diagnosed between January 1, 2003 and December 31, 2010 were identified from the TCR, which was established in 1979 to monitor the incidence and mortality rates of cancer in Taiwan [27]. Under the current system, the TCR records 97% of the cancer cases in Taiwan [27], and the quality of the TCR is comparable to other well-established cancer registries worldwide [28,29]. The morphology (M) codes of the International Classification of Diseases for Oncology, Field Trial Edition (ICD-O-FT) (for those diagnosed from January 1, 1996 to December 31, 2001) or the International Classification of Diseases for Oncology, Third Edition (ICD-O–3) (for those diagnosed after January 1, 2002) were used to identify ASC, adenocarcinoma (AC) and squamous cell carcinoma (SCC) cases. The M code is 8560/3 for ASC, 8140/3 for AC, and 8070/3 for SCC. The ICD codes to identify the sites of ASCs are presented in the Table in S1 Table.

The male to female (M/F) ratios for all ASCs and the cases by organ systems were calculated. The locations of ASCs were separated into three systems, including the female reproductive system, the respiratory system, and the alimentary system. The date of death for the ASC cases was determined by linking the TCR data to the national death database. Because the survival rates of ASC in the alimentary system were poor and most patients died within 3 years, we use log-rank test to calculate the median overall survival (OS) of ASCs instead of 3-yr or 5-yr OS rates for all systems combined, by each system separately, and by gender. We also performed survival analysis for patients diagnosed as AC, SCC or ASC in organs with frequent ASC. The Cox proportional hazards regression model was performed to estimate the hazard ratio (HR) and 95% confidence interval (CI) of death associated with system, age, and gender. This study was approved by the Institutional Review Board of Taipei Veterans General Hospital, Taiwan (IRB Number: 2014-03-005CC), and all clinical investigation has been conducted according to the principles expressed in the Declaration of Helsinki. The records and information of patients were anonymized and de-identified prior to analysis.

## Results

### Distributions of ASC by tumor site

The most common primary site of ASC was the respiratory system, followed by the alimentary system, and the female reproductive system (73.8% vs. 16.2% vs. 10%, respectively, Table 1).

**Table 1:** Distribution of ASC by system.

Tumor site			Age (y/o)	TNM stage (I+II / III+IV)	P-value
	Female reproductive system (n = 56)			29/27	0.004*
		Ovary (n = 8)	40.1±17.0	7/1	
		Uterus (n = 25)	53.5±6.6	12/13	
		Cervix (n = 11)	54.8±7.3	7/4	
		Breast (n = 12)	52.2±11.7	9/3	
	Respiratory system (n = 426)			139/287	
		Lung/bronchus (n = 426)	65.5±12.1	139/287	
	Alimentary system (n = 94)			24/70	
		Oropharynx (n = 16)	61.4±10.9	4/12	
		Esophagus (n = 24)	60.5±14.4	9/15	
		Stomach (n = 11)	69.9±12.8	10/1	
		Colon (n = 21)	58.8±297.8	5/16	
		Rectum/anus (n = 6)	71.3±9.8	3/3	
		Liver (n = 6)	69.2±10.4	5/1	
		Biliary tract (n = 10)	69.0±49.6	9/1	

*the statistical difference of TNM stages among different systems

With regard to the female reproductive system, the most common primary site of ASC was the uterus (44%), followed by the cervix (21%), breast (21%) and ovary (14%). For the alimentary system, the most common primary site of ASC was the esophagus (2.9%), followed by the colon (2.5%), oropharynx (1.9%), stomach (1.3%), liver (0.6%), and rectum/anus (0.6%). With regard to the TNM stage, ASCs in the alimentary system were associated with more advanced TNM stage (stage III and IV) compared with respiratory and female reproductive systems (74.5% vs. 67.4% vs. 48.2%, *P* = 0.004).

**Fig 1:**
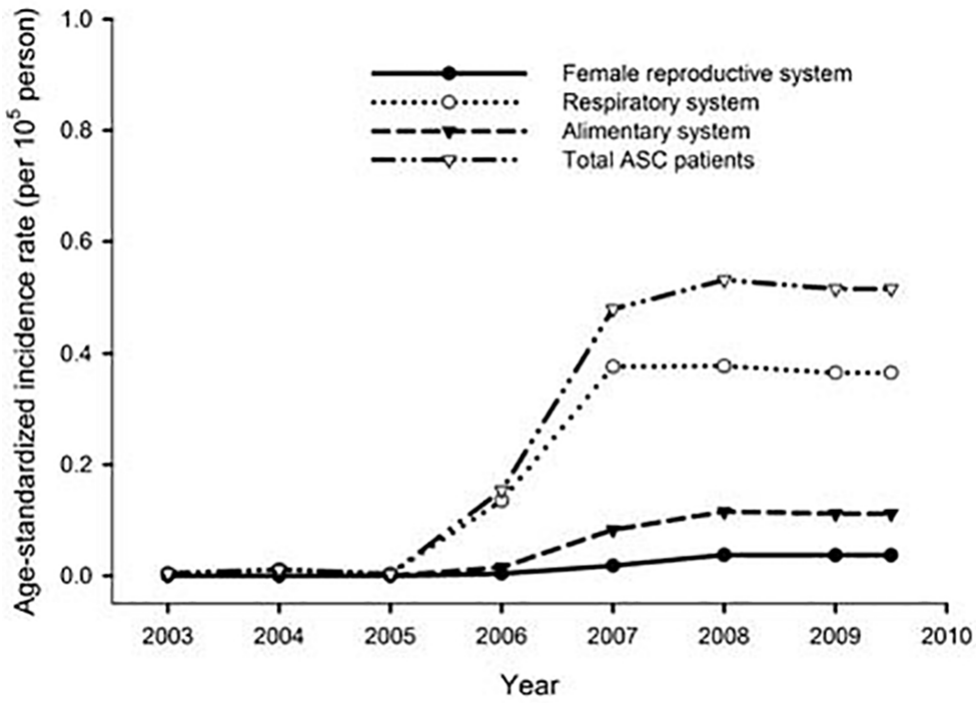
The age-standardized incidence rates of ASC according to different systems.

As shown in Fig 1, the age standardized incidence rates of ASC were low between year 2003 and 2005 and increased to a plateau after year 2007, which was highest in the respiratory system, followed by the alimentary system and the female reproductive system.

### Survival

**Fig 2:**
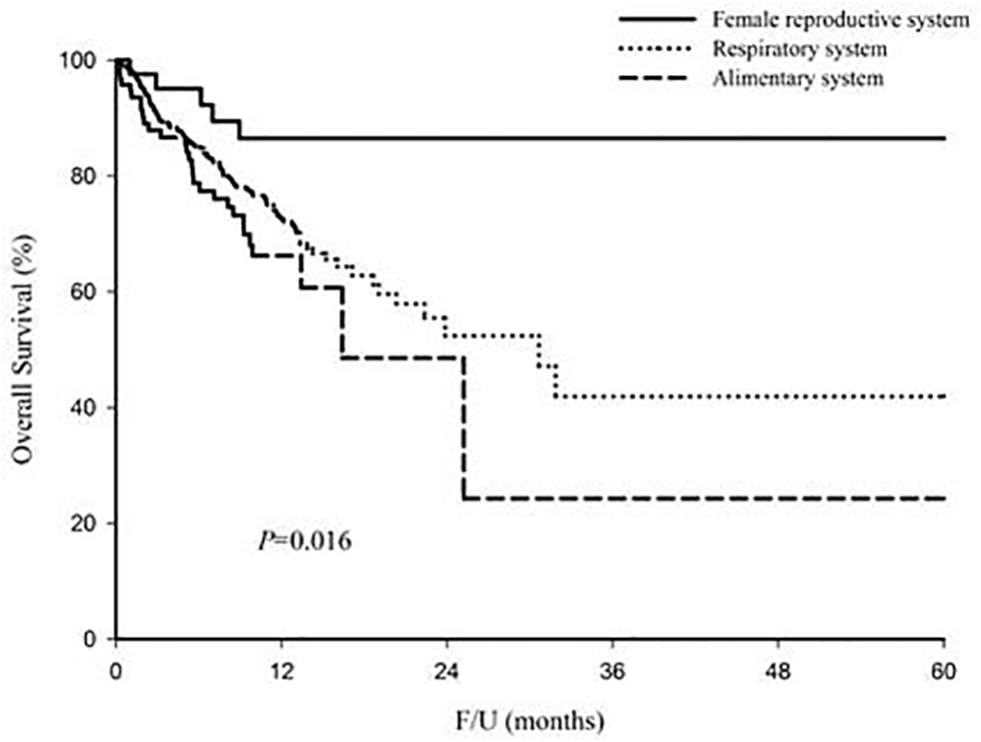
The overall survival were significantly higher in ASC patients with tumor located in the female reproductive system, followed by the respiratory system and alimentary system (*P* = 0.016).

As shown in Table 2 and Fig 2, the overall survival was significantly higher for cases involving the female reproductive system, followed by the respiratory and alimentary systems (*P* = 0.016). Because the follow-up time and survival was short in some patients, we calculate the median overall survival instead of 3-year or 5-year overall survival rate. The median overall survival were unable to calculate in some patients because less than 50% of patients died until the last time of follow-up. With regard to the respiratory system, the median overall survival was worse in males than females (22.4 vs. 31.8 months, *P* = 0.044). There was no significant difference in the median overall survival between males and females for cases involving the alimentary system.

**Table 2:** The median overall survival of ASC.

Tumor site			F/U (month)	Median overall survival (months)			
			Median	Overall	Male	Female	P-value for gender difference
	Female reproductive system (n = 56)		11.0	—	—	—	—
		Ovary (n = 8)	10.2	—	—	—	—
		Uterus (n = 25)	9.5	—	—	—	—
		Cervix (n = 11)	12.0	—	—	—	—
		Breast (n = 12)	14.0	—	—	—	—
	Respiratory system (n = 426)		9.8	30.7	22.4	31.8	0.044
		Lung/bronchus (n = 426)	9.8	30.7	22.4	31.8	0.044
	Alimentary system (n = 94)		8.8	16.4	16.4	25.2	0.302
		Oropharynx (n = 16)	10.1	—	—	6.1	0.424
		Esophagus (n = 24)	9.1	—	—	—	0.115
		Stomach (n = 11)	9.1	9.3	9.3	5.6	0.671
		Colon (n = 21)	10.3	—	—	—	0.639
		Rectum/anus (n = 6)	12.6	16.4	16.4	—	0.886
		Liver (n = 6)	2.7	—	—	1.9	0.157
		Biliary tract (n = 10)	8.2	25.2	—	25.2	0.430
		Oropharynx (n = 16)	11.0	—	—	—	—

The median overall survival were unable to calculate in some patients because less than 50% of patients died until the last time of follow-up.

### Risk factors of overall survival

As shown in Table 3, univariate analysis showed that age≧65, tumor located in the alimentary system, more advanced T and N categories, and a more advanced TNM stage were unfavorable prognostic factors of overall survival in ASC. Age, tumor location, T and N categories were included in a multivariate Cox proportional hazards model with forward logistics regression stepwise procedure to adjust for the effects of covariates. In the model, we demonstrated that age≧65 and more advanced T and N categories were independent unfavorable prognostic factors of overall survival in ASC.

**Table 3:** Survival analysis to assess the risk of death of patients with ASC.

		Univariate analysis			Multivariate analysis		
		HR	95% CI	P value	HR	95% CI	P value
Primary tumor sites							
	Female reproductive system	Ref					
	Respiratory system	3.08	1.352–7.017	0.007			
	Alimentary system	4.24	1.752–10.257	0.001			
Age (y/o)							
	<65	Ref			Ref		
	≧65	1.81	1.288–2.519	0.001	1.57	1.116–2.211	0.010
Tumor size							
	<4 cm	Ref					
	4–8 cm	1.88	1.227–2.873	0.004			
	>8 cm	3.32	1.795–6.130	<0.001			
Cell differentiation							
	Well	Ref					
	Moderate	1.36	0.320–5.737	0.680			
	Poor	2.64	0.636–10.980	0.181			
T category							
	T1	Ref			Ref		
	T2	2.37	0.898–6.273	0.081	2.14	0.808–5.686	0.125
	T3	7.39	2.879–18.953	<0.001	4.93	1.858–13.076	0.001
	T4	7.80	3.159–19.269	<0.001	4.81	1.852–12.509	0.001
N category							
	N0	Ref			Ref		
	N1	3.26	2.183–4.876	<0.001	1.86	1.187–2.924	0.007
	N2	1.12	0.534–2.347	0.233	1.18	0.556–2.481	0.673
	N3	13.78	5.314–35.755	<0.001	7.08	2.572–19.476	<0.001
TNM stage							
	I	Ref					
	II	1.00	0.373–2.656	0.992			
	III	2.82	1.451–5.484	0.002			
	IV	6.77	3.686–12.416	<0.001			

Ref: reference

### Comparison of the clinicopathological characteristics between cervical and respiratory tract

With regard to the clinicopathological characteristics, ASC located in the respiratory tract (including the lung/bronchus) were associated with an older age, more advanced T and N categories, and a more advanced TNM stage compared to ASC in the cervical tract (including the ovary, uterus and cervix). Univariate analysis showed that an older age, larger tumor size, more advanced T and N categories, and more advanced TNM stage were unfavorable prognostic factors of overall survival. Age, tumor size, T and N categories were included a multivariate Cox proportional hazards model with forward logistics regression stepwise procedure and demonstrated that an older age and a more advanced T category were independent unfavorable prognostic factors of overall survival (Table 4).

**Table 4:** Univariate and multivariate analysis of factors affecting the overall survival of the ASC located in the cervical and respiratory tract.

		Univariate analysis			Multivariate analysis		
		HR	95%CI	P-value	HR	95%CI	P-value
Age (years)							
	<65	Ref				Ref	
	≧65	2.01	1.372–2.939	<0.001		2.09	1.297–3.377
Tumor size (cm)							
	<4	Ref					
	4–8	1.98	1.240–3.160	0.004			
	>8	4.65	2.203–9.814	<0.001			
Cell differentiation							
	Well	Ref					
	Moderate	1.11	0.260–4.765	0.885			
	Poor	1.73	0.407–7.311	0.460			
T category							
	T1	Ref				Ref	
	T2	2.11	0.793–5.635	0.004		2.59	0881–7.636
	T3	4.68	1.649–13.297	<0.001		6.26	1.851–21.165
	T4	6.84	2.762–16.913	<0.001		7.71	2.772–21.430
N category							
	N0	Ref					
	N1	2.82	1.858–4.281	<0.001			
	N2	0.52	0.183–1.468	0.216			
TNM stage							
	I	Ref					
	II	1.34	0.501–3.566	0.241			
	III	2.11	1.045–4.275	<0.001			
	IV	5.70	3.081–10.529	<0.001			
Tumor location							
	Cervical tract	Ref					
	Respiratory tract	2.21	0.970–5.031	0.059			

Ref: reference

### Comparison among adenocarcinoma (AC), squamous carcinoma (SCC) and ASC in different organs

**Fig 3:**
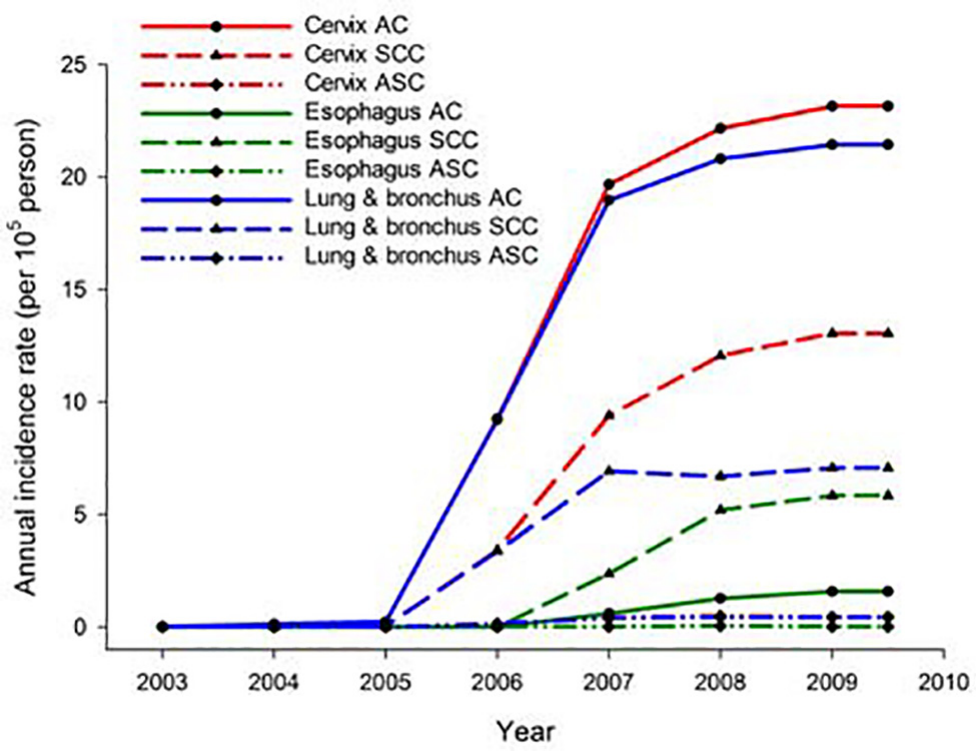
The annual incidence rates of AC, SCC and ASC in different organs.

We further compared the clinicopathological characteristics for 30,850 patients in the TCR database, who were diagnosed as either AC, SCC or ASC in the cervix (n = 238), lung/bronchus (n = 25,786), and esophagus (n = 4,826). As shown in Fig 3, the annual incidence rate of AC was higher than SCC and ASC in lung/bronchus and cervix; however, the annual incidence rate was highest for SCC, followed by AC and ASC in esophagus. The highest annual incidence of AC was located in the cervix, followed by the lung/bronchus and esophagus. The highest annual incidence of SCC was located in the cervix, followed by the lung/bronchus and esophagus.

In Table 5, most ASC, SCC and ASC were diagnosed at a more advanced stage in the esophagus and lung/bronchus, while most ASC, SCC and ASC were diagnosed at an earlier stage in the cervix. ASC was associated with a worse survival compared to AC and SCC in the esophagus, lung/bronchus and cervix. Multivariate analysis showed that tumor located in the esophagus, ASC histology, more advanced T and N categories were independent unfavorable prognostic factors of overall survival (Table 6).

**Table 5:** Comparison of the TNM stage at diagnosis and survival analysis among different histologic types of cancer in cervix, lung/bronchus, and esophagus.

		TNM stage (I+II / III+IV)		Overall survival	
			P-value	HR (95% CI)	P-value
Cervix (n = 238)			0.632		
	AC (n = 102)	77/25		Ref	
	SCC (n = 125)	94/31		0.68 (0.197–2.357)	0.545
	ASC (n = 11)	7/4		19.93 (8.216–48.334)	<0.001
Lung & bronchus (n = 25786)			<0.001		
	AC (n = 18,927)	3,084/15,843		Ref	
	SCC (n = 6,433)	1,281/5,152		1.51 (1.441–1.585)	<0.001
	ASC (n = 426)	139/287		2.52 (2.244–2.820)	<0.001
Esophagus (n = 4826)			<0.001		
	AC (n = 981)	158/136/282/405		Ref	
	SCC (n = 3,821)	333/809/1,325/1,354		1.32 (1.164–1.493)	<0.001
	ASC (n = 24)	9/15		2.69 (1.650–4.387)	<0.001

AC: adenocarcinoma; SCC: squamous cell carcinoma; ASC: adenosquamous carcinoma; Ref: reference

**Table 6:** Survival analysis to assess the risk of death of patients with adenocarcinoma, squamous cell carcinoma and adenosquamous carcinoma in the cervix, lung/bronchus, and esophagus.

		Univariate analysis			Multivariate analysis		
		HR	95% CI	P-value	HR	95% CI	P-value
Primary tumor sites							
	Cervix	Ref			Ref		
	Lung/bronchus	1.02	0.967–1.001	0.070	1.09	0.889–0.949	<0.001
	Esophagus	1.08	1.066–1.093	<0.001	1.13	1.106–1.152	<0.001
Histological type							
	AC	Ref			Ref		
	SCC	1.08	0.860–1.011	0.092	1.11	0.810–1.002	0.054
	ASC	1.04	1.026–1.050	<0.001	1.06	0.919–0.959	<0.001
Age (y/o)							
	<65	Ref			Ref		
	≧65	1.12	1.103–1.126	<0.001	1.10	1.085–1.121	<0.001
Tumor size (cm)							
	<4	Ref					
	4–8	1.00	0.992–1.017	0.511			
	>8	1.02	0.990–1.051	0.194			
Cell differentiation							
	Well	Ref					
	Moderate	1.01	0.977–1.011	0.469			
	Poor	1.01	0.969–1.007	0.211			
T category							
	T1	Ref			Ref		
	T2	1.10	0.899–0.929	<0.001	1.10	0.901–0.936	<0.001
	T3	1.02	0.961–0.994	0.009	1.09	0.894–0.938	<0.001
	T4	1.08	0.906–0.950	<0.001	1.15	0.867–0.918	<0.001
N category							
	N0	Ref			Ref		
	N1	1.09	0.900–0.931	<0.001	1.06	0.917–0.952	<0.001
	N2	1.12	0.872–0.907	<0.001	1.10	0.890–0.930	<0.001
	N3	1.19	0.812–0.877	<0.001	1.15	0.833–0.909	<0.001
TNM stage							
	I	Ref					
	II	1.10	0.899–0.928	<0.001			
	III	1.15	0.850–0.880	<0.001			
	IV	1.16	0.845–0.880	<0.001			

AC: adenocarcinoma; SCC: squamous cell carcinoma; ASC: adenosquamous carcinoma; Ref: reference

## Discussion

Using the TCR data, the age standardized incidence rate was low between year 2003 and 2005, and increased to a plateau after year 2007. The possible reasons include the improved quality of cancer registration, the increased awareness of ASCs by clinicians, and improvements in diagnostic technology. Because ASC is a rare histologic type in various organs, the low number of cases might influence the overall survival rate. Therefore, we use the TCR data in Taiwan to enroll additional patients, which might decrease selection bias and increase the reliability of our results. Our results showed that the overall survival was significantly higher in cases involving the female reproductive system, followed by the respiratory and alimentary systems (*P* = 0.016). Multivariate analysis showed that age≧65 and more advanced T and N categories were independent unfavorable prognostic factors of overall survival in ASC. To our knowledge, this is the first study comparing survival differences in ASC among different tumor locations.

With regard to the female reproductive systems, our results showed an excellent prognosis and more than 50% of patients survived until the last time of follow-up. Our breast ASC patients had a 100% 5-year OS rate, because breast ASC is usually low grade and associated with a favorable prognosis [2]. There are few case reports in the literature of ovarian or endometrial ASC [30,31], and the prognostic significance of ASC in the ovary or endometrium is still unclear. For cervical cancer, ASC was reported to be an independent poor prognostic factor compared with adenocarcinoma, especially in advanced stage cervical cancer [7]. Similar findings were observed in our study; with regard to cervical cancer, ASC was associated with a worse overall survival compared with AC and SCC (hazard ratio 19.93, 95% CI 8.216–48.334).

Regarding to the alimentary system, as shown in Fig 1 and Table 2, the overall survival was poor in the alimentary tract. However, because of the very rare incidence of ASC in the alimentary system, the small number of patients in the specific organs, such as stomach (n = 11), biliary tract (n = 10), rectum/anus (n = 6) and liver (n = 6) would affect the results. Our previous study of 7 cases of gastric ASC in a single institute also showed a poor prognosis with 3-year overall survival rates of only 28.6% [24]. The extremely poor survival of gastric and biliary tract ASC might be due to the aggressive tumor behavior and poor response to chemotherapy. Reports [32] show that gastric ASC does not respond to the use of 5-FU-based chemotherapy, such as TS–1, which might be observed when ASC originates in other organs. As a result, further standard chemotherapy still needs to be established for the management of ASC.

It is interesting why the tumor location of ASC is associated with overall survival in univariate analysis but not statistically significant in multivariate analysis. Only age and T and N categories were independent prognostic factors. We should be careful to explain the results. We also analyzed the clinicopathological characteristics between ASCs located in the cervical tract and respiratory tract. ASCs in the cervical tract were associated with a younger age and an earlier tumor stage than those in the respiratory tract. Age and T category were independent prognostic factors; however, the tumor location of ASC was not. It is possible that ASCs in the female reproductive system, including the breast and cervical tract, might be associated with palpable breast mass or vaginal bleeding, which could be detected by the patient herself more easily than ASCs in other organs. ASCs in the female reproductive system are diagnosed at an earlier stage and are associated with a better prognosis compared with ASCs located at other systems. As a result, tumor location of ASC is not an independent prognostic factor of overall survival.

Our results showed that while comparing with AC and SCC, ASC histology is an independent unfavorable prognostic factor in the cervix, long/bronchus and esophagus. As a result, ASC histology is associated with a more aggressive tumor behavior; different carcinogenesis and genetic mutations might be involved in ASC.

In lung ASC [33], the frequency of *EGFR* mutations was reported to be 33.3% in tumor specimens and which were significantly more frequent in women than men (44.4% vs. 25%) and in never-smokers than smokers (40% vs. 16.7%). Moreover, the objective response rate was 26.5%, and the disease control rate was 65.3% with *EGFR*-tyrosine kinase inhibitor (TKI) treatment, such as gefitinib or erlotinib. In our study, women had a significantly better 3-year overall survival with ASC of the respiratory system than did men, and this difference in survival between women and men in our population may be explained by the carcinogenesis of ASC. Among the 426 ASCs of the respiratory system, most patients (61.5%) were male. Smoking is a risk factor for lung cancer, and the prevalence of male smokers is higher than that of female smokers in Taiwan. As a result, we hypothesized that the gender difference in ASC of the respiratory system might be due to different carcinogenesis between males and females.

Our previous study of gastric ASCs has shown that the component of adenocarcinoma and squamous cell carcinoma in metastatic lymph nodes may influence prognosis. It would be interesting to ascertain whether there is any difference between males and females for adenocarcinoma and squamous cell carcinoma components in the primary tumor and metastatic lymph nodes. Further investigation of the adenocarcinoma and squamous carcinoma components in ASC in various organs might answer this question.

Recently, some studies have investigated the molecular pathogenesis of ASCs in the pancreas, cervix, and lung [7,33,34]. ASC pancreatic tumors have somatic mutations in Up-frameshift 1 (*UPF1*), which encodes an RNA helicase essential for a highly conserved RNA degradation pathway called nonsense-mediated RNA decay [33]. In cervical ASC [7], positive rates of immunohistochemical staining for *EGFR*, *PDGFRA*, and *VEGFR2* were found to be 43%, 100%, and 73.3%, which might predict the sensitivity of ASCs to specific anti-receptor tyrosine kinases (*RTK*) drugs. In addition, the frequency of *EGFR* mutations in lung ASC was reported to be 13.1%-33.3% [33,35], and *KRAS* mutations in pulmonary adenocarcinomas are resistant to EGFR tyrosine kinase inhibitor therapy [36]. As a result, both *EGFR* and *KRAS* mutation should be examined in lung ASCs, and the results could provide useful information for the targeted therapy of pulmonary ASCs.

Several limitations must be considered when interpreting the results of the present analysis. The number of ASC cases may have been underestimated. However, to our knowledge, the ASC cases included in the present study is the largest series to date, and we expect that our results will shed light on the study of ASC in the future.

In conclusion, the current article presents the first nation-wide cancer registry-based study of ASC. Our data showed that T and N categories and age were independent prognostic factors affecting overall survival. Further investigation of the carcinogenesis and treatment of ASC is required to improve the prognosis of this rare histologic type of cancer.

## Supporting Information

S1 Table ICD codes for identifying the sites of adenosquamous carcinoma. (DOC)
