# Supplementary material for: A Nation-Wide Cancer Registry-Based Study of Adenosquamous Carcinoma in Taiwan
Source: PLoS One. 2015 Oct 7;10(10):e0139748. doi: 10.1371/journal.pone.0139748 (PMC4596803; doi:10.1371/journal.pone.0139748)
Supplement: S1 Table — (DOC) [file pone.0139748.s001.doc]

S1 Table. ICD codes for identifying the sites of adenosquamous carcinoma.

| Site | ICD-O-3 | ICD-9 |
| --- | --- | --- |
| Oropharynx | C00-14 | 140-149 |
| Esophagus | C15 | 150 |
| Stomach | C16 | 151 |
| Small intestine | C17 | 152 |
| Colon | C18 | 153 |
| Rectum/anus | C19-21 | 154 |
| Liver | C22.0 | 155 |
| Biliary tract | C23-24 | 156 |
| Pancreas | C25 | 157 |
| Lung and bronchus | C33-34, C39 | 162 |
| Breast | C50 | 174-175 |
| Ovary | C56.9 | 183.0 |
| Uterus | C54-55 | 179, 182 |
| Cervix | C53 | 180 |
